# Unintentional guideline deviations in hospitalized patients with two or more antithrombotic agents: an intervention study

**DOI:** 10.1007/s00228-021-03185-y

**Published:** 2021-07-28

**Authors:** Renate C. A. E. van Uden, Marcel P. H. van den Broek, Ilse Houtenbos, Tessa C. C. Jaspers, Ankie M. Harmsze, Hylke J. Kingma, Diego A. M. Odekerken, Karina Meijer, Patricia M. L. A. van den Bemt, Matthijs L. Becker

**Affiliations:** 1 Pharmacy Foundation of Haarlem Hospitals, Haarlem, The Netherlands; 2 Department of Clinical Pharmacy, Spaarne Gasthuis Hospital, Haarlem/Hoofddorp, The Netherlands; 3 Department of Clinical Pharmacy and Pharmacology, University of Groningen, University Medical Center Groningen, Groningen, The Netherlands; 4 Department of Clinical Pharmacy, St Antonius Hospital, Nieuwegein/Utrecht, The Netherlands; 5 Department of Internal Medicine, Spaarne Gasthuis Hospital, Haarlem/Hoofddorp, The Netherlands; 6 Department of Clinical Pharmacy, Elisabeth-TweeSteden Hospital, Tilburg, The Netherlands; 7 Department of Cardiology, Spaarne Gasthuis Hospital, Haarlem/Hoofddorp, The Netherlands; 8 Department of Haematology, University of Groningen, University Medical Center Groningen, Groningen, The Netherlands

**Keywords:** Anticoagulants, Guideline adherence, Hospital medicine, Platelet aggregation inhibitors

## Abstract

**Purpose:**

Treatment schedules for antithrombotic therapy are complex, and there is a risk of inappropriate prescribing or continuation of antithrombotic therapy beyond the intended period of time. The primary aim of this study was to determine the frequency of unintentional guideline deviations in hospitalized patients. Secondary aims were to determine whether the frequency of unintentional guideline deviations decreased after intervention by a pharmacist, to determine the acceptance rate of the interventions and to determine the type of interventions.

**Methods:**

We performed a non-controlled prospective intervention study in three teaching hospitals in the Netherlands. We examined whether hospitalized patients who used the combination of an anticoagulant plus at least one other antithrombotic agent had an unintentional guideline deviation. In these cases, the hospital pharmacist contacted the physician to assess whether this deviation was intentional. If the deviation was unintentional, a recommendation was provided how to adjust the antithrombotic regimen according to guideline recommendations.

**Results:**

Of the 988 included patients, 407 patients had an unintentional guideline deviation (41.2%). After intervention, this was reduced to 22 patients (2.2%) (p < 0.001). The acceptance rate of the interventions was 96.6%. The most frequently performed interventions were discontinuation of an low molecular weight heparin in combination with a direct oral anticoagulant and discontinuation of an antiplatelet agent when there was no indication for the combination of an antiplatelet agent and an anticoagulant.

**Conclusion:**

A significant number of hospitalized patients who used an anticoagulant plus one other antithrombotic agent had an unintentional guideline deviation. Intervention by a pharmacist decreased unintentional guideline deviations.

**Supplementary information:**

The online version contains supplementary material available at 10.1007/s00228-021-03185-y.

## Introduction

In prescribing antithrombotic therapy, the risk of thrombotic events needs to be balanced by the risk of haemorrhage. For several indications, such as an acute coronary syndrome, the use of more than one antithrombotic agent is required [1–9]. If patients have multiple indications that require antithrombotic treatment, it is also possible that a combination of antithrombotics is indicated [1–9]. From a safety point of view, combining multiple antithrombotics is challenging since it is associated with an increased bleeding risk [10].

A nationwide Danish cohort study showed that the incidence rate of a non-fatal major haemorrhage was 2.3 per 100 patient years for vitamin K antagonist (VKA) monotherapy [10]. The combination of an anticoagulant, such as a VKA or a direct oral anticoagulant (DOAC) and one platelet inhibitor (i.e. double therapy), doubles the risk of major haemorrhage and the combination of an anticoagulant and two platelet inhibitors (i.e. triple therapy) increases this risk almost fourfold compared to VKA monotherapy [10, 11]. In a Dutch study on preventable medication-related hospital admissions, anticoagulants and platelet inhibitors contributed substantially, with percentages of 6.3% and 8.7%, respectively [12].

In patients using more than one antithrombotic agent, a substantial risk of medication errors arises. The combination of antithrombotics can be prescribed without a valid indication, or can be continued beyond the intended period of time. Most combinations of antithrombotic can be correct or incorrect, depending on indication(s) and duration of combination therapy. Some combinations are always incorrect, like the combination of a DOAC and low molecular weight heparin (LMWH). The risk of a new thrombotic event decreases over time for most indications [13–15]. Therefore, guidelines advise to use double and triple therapy for a limited time period varying from 1 week to more than 1 year, depending on the indication and patient characteristics [1–9]. Considering the bleeding risk, it is important that patients do not use (combinations of) antithrombotic medication longer than the intended period of time.

Several studies have evaluated the guideline adherence in patients using antithrombotic medication [16–19]. Proietti et al. and Lip et al. found that in patients with atrial fibrillation, 40.9–60.6% received adequate antithrombotic treatment, 6.8–21.7% were overtreated and 52.3–17.3% were undertreated and received no antithrombotic treatment [16, 17].

Warlé-van Herwaarden et al. and Minary et al. studied if patients with DAPT or a combination of anticoagulant therapy and antiplatelet therapy were overtreated. They found that 14–39.8% were overtreated with antithrombotic therapy [18, 19]. These studies showed that a considerable proportion of patients is under- or overtreated with antithrombotic therapy, thereby exposing patients to an increased bleeding risk or thrombotic risk [16–19]. These non-intervention studies, mainly focused on patients treated with antithrombotics for a specific indication, were executed in a small non-hospital setting and did not check whether guideline deviation was intentional or unintentional. Therefore, we conducted this study that checked in all admitted patients with more than one antithrombotic agent whether they had a possible guideline deviation. We checked not only if they had a deviation, but also if it was an intentional or unintentional deviation, and in case of an unintentional deviation, we gave a specific recommendation how to adjust the antithrombotic regimen adequately according to guideline recommendations.

The primary aim of this study was to determine the frequency of unintentional deviations in adherence to the relevant guidelines recommendations for the combination of an anticoagulant plus at least one other antithrombotic agent in hospitalized patients.

Secondary aims were whether the frequency of unintentional guideline deviations decreased after intervention by a pharmacist, the acceptance rate of the interventions, the type of interventions, whether there was a difference in unintentional deviations between hospitals, and to determine potential risk factors of unintentional guideline deviations.

## Methods

### Setting

This non-controlled prospective intervention study was conducted in three general teaching hospitals in the Netherlands. Patients were included in the Spaarne Gasthuis (Haarlem/Hoofddorp) between May 2018 and February 2019, in the St. Antonius Hospital (Nieuwegein/Utrecht) between February 2019 and May 2019 and in the Elisabeth Tweesteden Hospital (Tilburg) between July 2019 and January 2020. The study was initiated in the Spaarne Gasthuis and subsequently the St. Antonius Hospital and the Elisabeth Tweesteden Hospital participated in this study.

The St. Antonius Hospital and the Elisabeth Tweesteden Hospital both perform percutaneous coronary interventions (PCIs) and are referral centres for vascular surgery. The St. Antonius Hospital also performs coronary artery bypass graft surgery. The institutional review board of the Spaarne Gasthuis approved the study protocol. This study was additionally reviewed by the MEC-U Medical Ethics Committee (W18.213) which concluded that ethical approval and written informed consent was not required, as the study did not fall under the scope of the Dutch Medical Research Involving Human Subjects Act. The research was conducted in line with good clinical practice and Dutch privacy legislation.

### Study population

All patients of 18 years and older, admitted to the hospital using the combination of a DOAC with a LMWH or an anticoagulant with one or more platelet inhibitors were included. VKAs, DOACs and LMWHs, if prescribed in a therapeutic dosage, were included as anticoagulant. Therapeutic dosages of LMWH were nadroparin twice daily ≥ 2850 IU or once daily > 5700 IU, tinzaparin > 4500 IU per day or dalteparin > 5000 IU per day. Acetylsalicylic acid, clopidogrel, prasugrel and ticagrelor were included as platelet inhibitors. The sole combination of acetylsalicylic acid with dipyridamole was not included since this combination when started should be used continuously. When dipyridamole was used in combination with another antithrombotic agent, this combination was included. We focused on oral therapy, since intravenous therapy is not continued after discharge.

Apixaban ≥ 2.5 mg twice daily, edoxaban ≥ 30 mg per day, dabigatran ≥ 110 mg twice daily and rivaroxaban ≥ 10 mg per day were included as DOACs. Rivaroxaban 2.5 mg twice daily was not included since this dosage is not given for the same indications as the other oral anticoagulants are given. Phenprocoumon and acenocoumarol were included as VKAs. Patients were excluded if the indication for antithrombotic therapy was not noted in the hospital information system or was unknown by the physician. In the St. Antonius Hospital, patients admitted to the intensive care unit (ICU) were excluded because the antithrombotic therapy in this post cardiac surgery population is complex and the antithrombotic medication can change multiple times during the ICU admission. After discharge to a non-ICU ward, the patient was included.

### Study procedure

Patients were identified using the hospital information system EPIC (version 2015/2018, Epic Systems Corporation, Verona, WI, USA). Patients who met the inclusion criteria were selected by a validated algorithm and were presented on a patient list. The criteria needed to evaluate whether the prescribed antithrombotics were guideline based prescribed were not available for analysis by the algorithm. Therefore, all patients who had multiple antithrombotics prescribed were presented and not only the patients with a guideline deviation. There was no minimum duration how long patients needed to use the multiple antithrombotic therapy to be selected by the algorithm, since drug safety alerts were already shown to the prescriber.

The algorithm was used on top of regular medication surveillance. Regular medication surveillance included the surveillance of clinically relevant drug-drug interactions, over- or underdosing and contraindications, based on the G-standard, a national database maintained by the Royal Dutch Association for the Advancement of Pharmacy (KNMP). In two of the three hospitals, the medication surveillance system did not warn the clinicians in case of (potentially unintended) combinations of antithrombotic drugs.

After regular medication surveillance, the patients on the patient list were reviewed on a daily basis by the hospital pharmacist (resident) on duty for medication surveillance that day. The patient’s medical record was checked for relevant indications, for the intended duration of the antithrombotic therapy and for intentional guideline deviations. Based on this information, the pharmacist assessed whether the therapy was in line with current guidelines or whether the physician (intentionally or possibly unintentionally) deviated from them. The European guidelines from the European Society of Cardiology (ESC), the European Association for Cardio-Thoracic Surgery (EACTS), European Respiratory Society (ERS) and the European Society for Vascular Surgery (ESVS) were used [1–9]. Since there is no European guideline for cerebrovascular accident available, the Dutch national guideline was used [20]. Most of these guidelines focus on one condition, for instance, atrial fibrillation or NSTEMI. Some guidelines also provide guidance on what to do when a patient already uses antithrombotic therapy for another condition, for instance, the NSTEMI guideline [8]. The recommendations in the guidelines are often consistent with each other, but not all combinations of indications are mentioned. There is no guideline on antithrombotic therapy that sums up all possible combinations and intended durations. We refer to our previous paper that explains most of the possible combinations and indications of antithrombotic therapy [21].

If an unintentional guideline deviation was suspected, the pharmacist contacted the treating physician, in most instances by phone, and discussed the antithrombotic medication. If the deviation was unintentional, a recommendation how to adjust the antithrombotic regimen adequately in order to meet the guideline recommendations was provided by the pharmacist.

The day after the intervention, the pharmacist checked whether the antithrombotic medication had been adjusted. All interventions were reviewed by a second pharmacist when the data were analysed. If the second pharmacist did not agree with the first pharmacist, they discussed the intervention in order to reach consensus.

Before the start of the study, all pharmacists were trained with respect to the indications for antithrombotic treatment and the duration of antithrombotic therapy, according to the guidelines. During the course of the study, the investigators were available for the other pharmacists for questions about the antithrombotic therapy. In the Spaarne Gasthuis, a 1-h training on antithrombotic therapy was given to all new physicians at start of employment. This training was implemented several years before the start of the study. In the St. Antonius Hospital, all new staff who have prescription rights had to complete an e-learning on antithrombotic therapy. This training was implemented before the start of the study. In the Elisabeth Tweesteden Hospital, no specific training on antithrombotic therapy was provided to prescribers before or during the study. No other training was provided to prescribers during the study.

### Outcome measures

The primary outcome was the frequency of unintentional guideline deviations for the combination of an anticoagulant plus at least one other antithrombotic agent. Secondary outcomes were the difference in unintentional guideline deviations before and after intervention, the acceptance rate of the interventions by a pharmacist, the type of intervention for the unintentional deviation and the frequency of unintentional deviations per hospital. As potential risk factors older age (over 70 years of age), gender and cardiology versus non-cardiology wards were analyzed.

### Statistical analysis

Descriptive statistics were used to determine the frequency of unintentional guideline deviations. The difference before and after intervention was analyzed using a McNemar test. A one-way ANOVA test was used to analyze whether there was a difference in unintentional guideline deviations between the three hospitals. The association between possible risk factors and unintentional guideline deviation was analyzed using univariate logistic regression. The potential risk factors were analyzed for the patients using double or triple therapy only, since the combination of a DOAC with LMWH is always unintentional. Analyses were performed using IBM SPSS statistics for Windows, version 24.0 (IBM Corp. Armonk, NY). A p value below 0.05 was considered statistically significant.

## Results

**Fig. 1:**
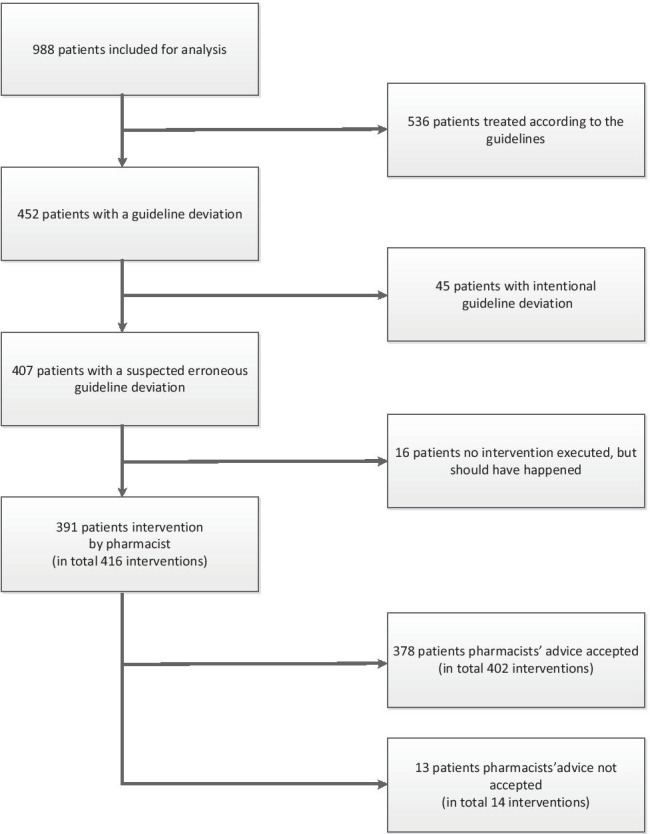
Patient flowchart

A total of 996 patients met the inclusion criteria for our study. Eight patients were excluded because the indication for antithrombotic therapy was unknown. We included 988 patients in our study (Fig. 1; Table 1). Of these, 635 (64.2%) were male, median age was 74 (interquartile range 69–81) and 516 (52.2%) patients were admitted to a cardiology ward.

**Table 1:** Patient characteristics

Characteristic	All patients n = 988
Male sex	635 (64.2)
Age, years	74 [69–81]
Type of antithrombotic therapy	
• DOAC + LMWH	182 (18.4)
• Anticoagulant + one antiplatelet agent	681 (68.9)
• Anticoagulant + DAPT	125 (12.7)
Used anticoagulant^a^	
• DOAC	525 (53.1)
• VKA	259 (26.2)
• LMWH^b^	204 (20.6)
Admission ward	
• Cardiology	516 (52.2)
• Neurology	39 (3.9)
• Surgery	190 (19.2)
• Other	243 (24.6)

Results are presented as median [interquartile range] or as number of patients (%)

*DOAC* direct oral anticoagulant, *VKA* vitamin K antagonist, *LMWH* low molecular weight heparin, *DAPT* dual antiplatelet therapy

^a^When a combination of a DOAC and LMWH was used this was scored as a DOAC user

^b^Therapeutic dosage of LMWH

An unintentional guideline deviation was observed in 407 of the 988 patients (41.2%). An intentional guideline deviation was observed in 45 patients (4.6%). After intervention, 22 of the 988 patients (2.2%) had an unintentional guideline deviation (p < 0.001; Table 2). In 391 of the 988 patients, 416 interventions were performed, of which 402 interventions were accepted by the physician (96.6%). In 13 patients, the recommendation to adjust the antithrombotic therapy was not adopted by the physician. In 16 patients, erroneously no intervention was performed by the first pharmacist. The most frequently performed interventions were discontinuation of an LMWH when used in combination with a DOAC (198 of the 416 interventions, 47.6%) and discontinuation of the antiplatelet agent when there was no indication for the combination of an antiplatelet agent and an anticoagulant (163 of the 416 interventions, 39.2%) (Tables 2 and 3).

**Table 2:** Deviations from guideline in 988 patients using more than one antithrombotic agent

	Number of patients with an unintentional deviation before intervention (%)	Number of patients with an unintentional deviation after intervention (%)	P value
Unintentional deviation	407 (41.2)	22 (2.2)	p < 0.001
• Double therapy	185 (18.7)	19 (1.9)	
• Triple therapy	40 (4.0)	3 (0.3)	
• DOAC + LMWH	182 (18.4)	0 (0.0)	

*DOAC* direct oral anticoagulant, *LMWH* low molecular weight heparin

**Table 3:** Performed interventions in patients using double or triple therapy

Recommendation	Number of interventions double and triple therapy (%) n = 806 patients 234 interventions
Stop LMWH^a^	26 (11.1)
Stop platelet inhibitor	163 (69.7)
Switch platelet inhibitor^b^	23 (9.8)
Other	22 (9.4)

*LMWH* low molecular weight heparin

^a^This could mean stopping LMWH in combination with a DOAC and platelet inhibitor or stopping therapeutic LMWH in combination with a platelet inhibitor or DAPT

^b^Most often switching from ticagrelor or prasugrel to clopidogrel was recommended. Double or triple therapy with ticagrelor or prasugrel increases the bleeding risk compared to clopidogrel. Therefore, the guideline recommends not to use ticagrelor or prasugrel as part of double or triple therapy

There was a statistically significant difference in frequency of unintentional guideline deviations between the hospitals (p < 0.001). In hospital A, 222 of the 446 patients (49.8%) had an unintentional guideline deviation; in hospital B, 80 of the 315 patients (25.4%); and in hospital C, 105 of the 227 patients (46.3%).The combination of a DOAC with an LMWH occurred in hospital A in 122 of the 446 patients (27.4%), in hospital B in 15 of the 315 patients (4.8%) and in hospital C in 45 of the 227 patients (19.8%).

On non-cardiology wards, there were significantly more unintentional guideline deviations for double and triple therapy (46.7%) than on cardiology wards (17.1%) (OR 4.25 [3.07–5.88]) (Table 4). Women more often had an unintentional guideline deviation for double or triple therapy (34.8%) than men (24.5%) (OR 1.65 [1.20–2.27]). There was no difference in unintentional guideline deviations in patients over 70 years (27.8%) versus patients up to 70 years old (28.2%) (OR 0.90 [0.73–1.43]). Although no intervention had taken place during admission for 16 patients, in seven patients (44%), the therapy was adjusted correctly by the physician before the patients’ discharge.

**Table 4:** Risk factors before intervention in patients using double and triple therapy

Unintentional deviation before intervention	Double or triple therapy (n = 806) Unintentional deviation/all patients	Percentage of unintentional deviations	Odds ratio 95% confidence interval
Ward			-
• Cardiology	87/510	17.1%	
• Neurology	9/15	60.0%	
• Surgery	29/101	28.7%	
• Other	100/180	55.6%	
Ward			
• Cardiology	87/510	17.1%	Ref.
• Non-cardiology	138/296	46.7%	4.25 [3.07–5.88]*
Sex			
• Male	132/539	24.5%	Ref.
• Female	93/267	34.8%	1.65 [1.20–2.27]*
Age			
• ≤ 70 years	70/248	28.2%	Ref.
• > 70 years	155/558	27.8%	0.90 [0.73–1.43]

^*^p < 0.001

## Discussion

In our study, 41.2% of the patients had an unintentional guideline deviation for the prescribed combination of antithrombotic therapy. The proportion of patients who had an unintentional deviation was reduced to 2.2% after intervention by a pharmacist.

An example of the most frequently occurring unintentional guideline deviation was when a patient who already used an antiplatelet agent was diagnosed with an indication requiring anticoagulant therapy. For instance, a patient who suffered from an ischemic cerebrovascular accident in the past for which clopidogrel is prescribed and is currently diagnosed with atrial fibrillation. As long as the patient is treated with an anticoagulant, clopidogrel should be withheld.

On cardiology wards, less unintentional deviations occurred compared to non-cardiology wards. This meets the expectations since cardiologists prescribe this type of medication more often and are therefore more familiar with the guidelines and more aware of the risks. In women, more unintentional deviations were found compared to men; however, an explanation for this observation could not be found. There was a difference between the hospitals in the frequency of unintentional deviations. In hospital B, the erroneous combination of DOAC with an LMWH was less often prescribed than in the other hospitals. A possible explanation for this could be that, in hospital B, the medication surveillance system did warn the clinicians in case of (potentially intended) combinations of antithrombotic drugs. That being said, a considerable number of patients in hospital B got the combination of a DOAC with an LMWH and other erroneous combinations. Therefore, evaluating the antithrombotic medication in patients using more than one antithrombotic agent is required even when the medication surveillance warns for duplicate medication because the results show that alerts are overridden.

Overriding medication safety alerts and alert fatigue are known problems [22, 23].

Considering the fact that a significant number of unintentional guideline deviations were observed in all three hospitals, we expect that this is a general problem. A better evaluation of patients who use more than one antithrombotic agent by both physicians and pharmacists is necessary. Education in antithrombotic therapy for physicians and pharmacist should focus more on when usage of more than one antithrombotic agent is indicated as well as on more awareness of the use of DOACs in order to avoid the combination of DOACs with LMWH prophylaxis.

The frequency of unintentional guideline deviations in our inpatient population was comparable to that reported in a previous study in outpatients. Warlé-van Herwaarden et al. found that 19 of the 82 (23.2%) patients who used a VKA plus an antiplatelet agent had an intentional or unintentional guideline deviation [18]. In our study, we found a proportion of 33.5% (intentional plus unintentional deviation OAC + one platelet inhibitor). Larock et al. found that in a Belgian hospital that 51 of the 106 (48.0%) hospitalized patients who used a combination of a DOAC and one antiplatelet agent had a guideline deviation versus 33.6% in our study [24]. But in Larock’s study, it was not specified which guidelines were used. For example, they classified double therapy during 6 months after a PCI as guideline based, while the European Society of Cardiology guideline recommend that double therapy can be continued until 12 months after PCI [4]. Therefore, the results are not comparable with our results. Minary et al. found that in a French university hospital, 37 out of 93 (39.8%) patients with atrial fibrillation who were over 75 years old and used a VKA in combination with an antiplatelet agent, the antiplatelet agent should have been stopped [19]. These results are not completely comparable with our results because they only included patients over 75 years old with the indication of atrial fibrillation.

The high number of deviations reflects that a substantial number of patients are prescribed more antithrombotic therapy than necessary, resulting in an increased bleeding risk. Van Rein et al. found that the major bleeding incidence increases considerably when more than one antithrombotic agent is used [10]. Therefore, unintentional continuation of antithrombotic therapy should be prevented in order to decrease the bleeding risk. Proiettie et al. found that in 2535 patients with atrial fibrillation, 40.9% of these patients were adequately treated with antithrombotic therapy and 6.8% of the patients were overtreated [16]. Guideline non-adherent patients had higher rates for all-cause death (8.9 versus 3.4%, p = 0.007) versus guideline adherent patients [16]. Lip et al. found in 2634 patients in the EORP-AF cohort that 60.6% of the patients were guideline adherent, 17.3% were undertreated and 21.7% were overtreated [17]. Overtreatment was associated with a significant higher number of all-cause mortality, any thromboembolism and the composite endpoint of cardiovascular death and any thromboembolism or bleeding when compared with patients with guideline-adherent antithrombotic pharmacotherapy [17].

Our study has some strengths and limitations. The main strength is that our study was a prospective intervention study. We checked not only whether the therapy deviated from guideline recommendations, but also whether recommendations from a pharmacist improved guideline adherence. In addition, we differentiated between intentional and unintentional deviations. To the best of our knowledge, this is the first study that also analysed patients who received a combination of an LMWH and a DOAC. While many studies have shown the benefits of DOACs over VKAs, they do not mention the risk of erroneously combining an LMWH with a DOAC [25, 26]. Another strength was that the study was performed in three hospitals. In all three hospitals, a considerable number of patients had an unintentional guideline deviation. Therefore, we believe that unintentional guideline deviations may occur in more hospitals.

A potential limitation of our study is the lack of a control group. Therefore, we do not know whether the antithrombotic therapy would have been corrected during follow-up if no intervention had taken place. In 16 patients with a suspected unintentional guideline deviation, no evaluation with the prescribing physician took place. In seven of these patients, the antithrombotic therapy was adjusted in line with the guideline recommendations during admission without intervention from a pharmacist. Nevertheless, for the other nine patients, the deviation was not corrected. We did not study the effect on the incidence of bleeding and thrombotic events. It is to be expected that better guideline adherence will result in a lower frequency of these events.

In conclusion, this study showed that a significant number of hospitalized patients who used the combination of an anticoagulant plus at least one other antithrombotic agent had an unintentional guideline deviation. This frequency decreases significantly after intervention from a pharmacist. We strongly recommend to implement an evaluation of patients using anticoagulant combined with at least one other antithrombotic agent to improve medication safety for admitted patients.

## Supplementary Information

Below is the link to the electronic supplementary material.

Supplementary file1 (DOCX 15 kb)

## Data Availability

The datasets used and analyzed during the current study are available from the corresponding author on reasonable request.
