# Supplementary material for: Unintentional guideline deviations in hospitalized patients with two or more antithrombotic agents: an intervention study
Source: Eur J Clin Pharmacol. 2021 Jul 28;77(12):1919–26. doi: 10.1007/s00228-021-03185-y (PMC8585825; doi:10.1007/s00228-021-03185-y)
Supplement: Supplementary file 1 — Supplementary file1 (DOCX 15 kb) [file 228_2021_3185_MOESM1_ESM.docx]

Table

| **Indication** | **Guideline** |
| --- | --- |
| **Antiplatelet therapy** |  |
| Cerebrovascular accident or Transient Ischemic Attack  Carotid stenosis | 2019 FMS Herseninfarct en hersenbloeding^18^  (Cerebral infarction and cerebral haemorrhage) |
| Coronary artery disease/  Stable Angina | 2013 ESC Guidelines on the management of stable coronary artery disease^1^ |
| Acute coronary syndrome (NSTEMI, STEMI, iAP) or PCI / CABG in stable CAD setting | 2017 ESC focused update on dual antiplatelet therapy in coronary artery disease^2^  2020 ESC Guidelines for the management of  acute coronary syndromes in patients  presenting without persistent ST-segment  elevation^8^ |
| TAVI procedure  Aortic bioprosthesis or valve sparing surgery/  Surgical implantation of a mitral or tricuspid bioprothesis  Surgical mitral or tricuspid valve repair | 2017 ESC/EACTS Guidelines for the management of valvular heart disease^3^ |
| Peripheral arterial disease  Carotid stenosis stenting  Peripheral percutaneous revascularization  Below the knee bypass with prosthetic graft  Venous bypass | 2017 ESVS Guidelines on the diagnosis and treatment of peripheral arterial diseases^4^ |
| **OAC** |  |
| Non-valvular atrial fibrillation or atrial flutter | 2016 ESC Guidelines for the management of atrial fibrillation^6^ |
| Venous thromboembolism | 2019 ESC Guidelines for the diagnosis and  management of acute pulmonary embolism  developed in collaboration with the European  Respiratory Society (ERS)^9^ |
| Mechanical valve | 2017 ESC Guidelines for the management of valvular heart disease^3^ |
| Biological mitral of tricuspidal valve < 3 months (implantation or reconstruction) | 2017 ESC/EACTS Guidelines for the management of valvular heart disease^3^ |
| **Double therapy** |  |
| Atrial fibrillation, NSTEMI and PCI | 2020 ESC Guidelines for the management of  acute coronary syndromes in patients  presenting without persistent ST-segment  elevation^8^ |
| **Triple therapy** |  |
| Atrial fibrillation, NSTEMI and PCI < 1 week | 2020 ESC Guidelines for the management of  acute coronary syndromes in patients  presenting without persistent ST-segment  elevation^8^ |

FMS= Federatie Medisch Specialisten (Federation of Medical specialists)

ESC = European Society of Cardiology

EACTS= European Association for Cardio-Thoracic Surgery

ESVS= European Society of Vascular Surgery

PCI= Percutaneous coronary intervention

CABG= Coronary artery bypass grafting

TAVI=Transcatheter aortic valve implantation

CAD= Coronary artery disease

NSTEMI= non ST segment elevation myocardial infarction

STEMI= ST segment elevation myocardial infarction

iAP= instable angina pectoris
